# J. D. Chevalier

**Published:** 1852-07

**Authors:** 


*Mr. J. D. Chevalier.*—This gentleman, so well known to the profession, appears to be determined to meet all demands on his skill and ingenuity. He is ever coming out with something new and also useful. At one time we have a head-rest, at another a lathe, and when we thought we had headed him with drawings of Dr. Baxter's Plugging Forceps, he sends us an old cut of one on a similar style.

We would assure Mr. Chevalier, however, that Dr. Baxter's with his numerous points, embrace more than the drawing which he sent, and at the same time we have good reason to suppose, that Dr. Baxter had never seen one of Mr. Chevalier's make. We have no idea that Dr. Baxter, would wish to detract a particle from Mr. Chevalier's improvement, but is willing that the Profession should enjoy all the advantages they can from what he has done.

We do not recollect of ever having seen one of Mr. Chevalier's make, and as yet, only the one of Dr. Baxter, which he first modelled out of an old pair of forceps for his own use. Mr. Reese, of this city, is however, making a few to supply the present demand.

